# About Rheumatism

**Published:** 1915-11-13

**Authors:** 


### THE EDITOR'S LETTER-BOX.

### ABOUT RHEUMATISM.

*To the Editor of* The Hospital.

Sir,—One is so accustomed to reading concise yet comprehensive and informative articles in The Hospital that your recent account of "Rheumatism in its Professional and Popular Aspects" (The Hospital, October 30, 1915) came as something of a painful surprise to one constant reader at least. Surely his opening sentence might have warned the writer thereof that at the present stage of progress the *clientèle* of an up-to-date journal of administrative and practical medicine require a careful *résumé* of our knowledge of such an important subject as "rheumatism," rather than a prologue devoid of useful information, an incomplete account of rheumatic manifestations, and a few odd remarks on the origin of the term "rheumatism." Really your writer on rheumatism seems to have had no other thought than to prove the truth of his first few words: "Of making of books and papers on rheumatism there is apparently no end."

Now, Sir, having said so clearly that I did not like this article, may I, as a true believer in your paper, state my reasons a little more definitely? And at once I will take exception to the apparent distrust of the average practitioner of medicine displayed by your author, who in one place writes as if doctors as a rule do not know their work well enough to appreciate the dangers of acute rheumatism in childhood.

The actual words used are: "If it were a universal professional rule to point out to the parents whenever a rheumatic tendency is discovered the risks which the apparently slight illnesses of their children involve, something would be gained." Ten or fifteen years ago there might have been ground for such an obvious aspersion on professional standards; to-day the heart dangers of "rheumatic fever" in early life are as well known to general practitioners as are the dangers of apoplexy or the vagaries of "specialists."

On the one point about which a scientific thinker on the subject might have given us enlightenment—causation—there is not vouchsafed a helpful word. The only conclusion arrived at apparently is that in regard to the relationship between acute and chronic forms of rheumatism "so long as the cause of these chronic conditions is unknown, we cannot confidently say that it is certainly not identical with the equally unknown cause of rheumatic fever." Why, indeed, this tortuous attempt to bring together two sets of conditions which modern physicians are inclined to think have nothing to do with each other except a common misnomer? We do not know the cause of cancer, neither do we know why sunspots trouble the surface of our great luminary; but is any useful object to be attained by stating it as a proposition that our ignorance in both directions prevents our saying that the causes of cancer and sunspots are not identical?

As to the professional aspects of rheumatism many of us would have eagerly welcomed reference to various modern treatments, particularly in regard to vaccines and phylacogens; if various results reported can be repeated at will the last-named bacterial preparations will prove to be a blessing indeed, but practitioners require an authoritative lead before they can go ahead with them. Then, again, the differential diagnosis of some gouty conditions, fibrositis, and neuritis in relation to acute and chronic rheumatism offer many difficulties which a responsible writer might have endeavoured to lessen for the benefit of officials and doctors working at institutions for the sick, such problems as those under notice being of exceptional importance to them all at this season.

Whilst as to the popular aspects of the question, reassurance might have been suggested as to the best endeavours of medical investigators to elucidate the problems of rheumatism, and some indication given of the progress that has already been made. Our present understanding of fibrositis—to mention a single point of advance—alone has enabled relief to be given on scientific lines to innumerable sufferers who in former times would probably have failed to get the right treatment. In a word, the public might reasonably have been reassured that modern medicine offers far more opportunities of assistance to those victimised by rheumatism than ever before.—I am, yours, etc.,

An Average Practitioner.

Harley Street, W., November 9, 1915.

